# Gold (III) adsorption from dilute waste solutions onto Amberlite XAD7 resin modified with L-glutamic acid

**DOI:** 10.1038/s41598-019-45249-1

**Published:** 2019-06-19

**Authors:** Maria Mihăilescu, Adina Negrea, Mihaela Ciopec, Corneliu Mircea Davidescu, Petru Negrea, Narcis Duţeanu, Gerlinde Rusu

**Affiliations:** 1 Politehnica University of Timisoara, Faculty of Industrial Chemistry and Environmental Engineering, 2 Piata Victoriei, RO 300006 Timisoara, Romania; 2 Politehnica University of Timişoara, Research Institute for Renewable Energies, 138 Gavril Muzicescu, RO 300501 Timisoara, Romania

**Keywords:** Chemical engineering, Chemical engineering

## Abstract

The main purpose of this paper was to obtain a material with efficient adsorbing properties and selectivity, to recover the gold (III) from residual diluted solutions resulted from the electroplating process. In this regard, a material was obtained by physico-chemical functionalization of a chemically inert support with functional groups of nitrogen and carboxyl. As a source of functional groups glutamic acid was used, and Amberlite XAD7 type acrylic resin was used as solid support. In order to establish the mechanism of the adsorption process, kinetic, thermodynamic and equilibrium studies were performed. The maximum adsorption capacity of the material has been established, and a gold (III) recovery process has been proposed using thermal decomposition of the exhausted adsorbed material. Main objective of this study was to evaluate an environmental friendly adsorbent material to recover gold from secondary industrial sources.

## Introduction

Due to their specific physical and chemical properties, precious metals are widely used in many areas, such as in the electrical and electronics industry, in various chemical processes, in the manufacture of catalysts, in the manufacture of corrosion resistant materials, and in the production of jewellery^1–5^. Over the last four decades, considerable amounts of gold have been used to produce electrical and electronic systems, because of their excellent electrical conductivity, low contact electrical resistance and remarkable corrosion resistance, being suitable for use in various connections^6,7^.

It is also known that historically, precious metals were and also have remained important in the manufacture of coins, being known worldwide as currency forms, according to ISO 4217^2^. As a result of the continued decline in gold quality and high demand on the market, its recovery becomes crucial and inevitable^8,9^.

The recovery process is effective if the recovery cost is much lower than the value of the precious recovered metal. In addition, restrictions imposed by environmental authorities on waste disposal require economic viability and green technologies^6,8^.

Mechanical, pyro-metallurgical, hydrometallurgical separation and bio-metallurgical technologies have been widely used for the recovery of gold from secondary sources^10–13^. Gold was mainly recovered from ores through the cyanide process^14–17^, a pollutants generating method with a significant negative impact on the environment; a worldwide controversial; other gold recovery agents are: ammonium thiosulphate^18^, royal water^19^, thiourea^20^, tiosulfates^21^, ammonia, iodine, natural organic acids, bromine, sodium sulphide^22^, and so on. Processes employing these chemical reagents are separation and purification processes (such as cementations, reduction, adsorption, coagulation, solvent extraction, ion exchange, gravity separation, ionic flotation, and so on^6^).

Some of the materials used for gold recovery, with adsorbent properties are: active carbon^23^, mesoporous absorbents^24^, chitosan or chemically modified oxides by functionalization^25,26^.

In this study, the obtained material has selective, relatively inexpensive, environmental friendly adsorbent properties and can be used with good results for recovering gold from dilute solutions which resulted as by-products from industrial processes. Gold was chosen because of its spread use at industry level but leads at resource exhaustion along with gold cost increase. Thus, it’s important to recover and reuse gold from industrial residual solutions. Thus, the use of the Amberlite XAD7 type resin as a support, and the functionalization of its surface with nitrogen and carboxyl groups using L - glutamic acid, can be an effective solution.

## Material and Methods

### Functionalized polymers preparation

To obtain the adsorbent material, 0.1 g of L-glutamic acid extractant (puriss., 99.0%, Merck, Germany) was weighed, over which 25 mL of acidulated DI water was added to obtain its dissolution. The dissolved extractant was contacted with 1 g of Amberlite XAD7 support (20–60 mesh, Sigma-Aldrich, Merck) in a solid: liquid ratio of 0.1 g: 25 mL. For functionalization, the support and the extractant were brought into contact for 24 hours, and then were dried in the oven (Nitech) for 24 hours at 323 K.

### Material characterization

Obtained material was analysed by X-ray energy dispersion (EDX) using a Quantum FEG 250 scanning electron microscope, and Fourier Transformed Infrared spectroscopy (FTIR) using a Bruker FT-IR spectrometer Platinum ATR-QL Diamond, in the range 4000–400 cm^−1^. Adsorbent material obtained after Amberlite XAD7 functionalization was dried for 24 h; afterwards, several granules of modified adsorbent were glued on the carbon adhesive disks and fixed on the stabs. These stabs were further used for data collection.

### Gold adsorption experiments onto Amberlite XAD7-glutamic acid

The pH of the solutions is a variable with a significant effect on the affinity of the material for a particular ion. This influence of pH is related to the form of the metal ions in the solution as well as the functional group of the extractant. Due to this, it was studied the influence of pH on the adsorption process of gold (III) on the obtained material, ranging pH from 2 to 14 to an initial concentration of Au (III) of C_0_ = 5 mg/L, using 0.1 g of adsorbent material, 1 h time contact and temperature 298 K. The pH of the solution was measured using the CRISON MultiMeter MM41 pH-meter.

Effect of contact time and the temperature of the adsorption process are other important factors for assessing the affinity of the material for Au(III) ions. To determine the influence of contact time and temperature on the adsorption capacity of the functionalized material, it was accurately weighed 0.1 g of material over which 25 mL of Au (III) concentration of 5 mg/L was added. The samples were shaken at 200 rpm for 15, 30, 45, 60, 90 and 120 minutes in a Julabo SW23 water bath with thermostatic and shaking control. All experiments were carried out and at different temperatures (298 K, 308 K and 318 K).

To determine the equilibrium concentration and effect of Au(III) initial concentration on material adsorption capacity, it was proceeded as follows: A residual solution of industrial cyanide baths was treated with HCl (37 wt.%, Sigma Aldrich) and HNO_3_ 63.013 wt.%, Chem Spider) to obtain a solution containing 2 g Au (III)/L. Further, by dilution of this stock solution was prepared a solution of 100 mg Au (III)/L; by further dilution Au (III) solutions of different concentrations (5, 10, 25, 50, 75, and 100 mg/L) were prepared. Absorptions experiments were performed on the produced functionalized material.

Adsorption studies were carried out at pH, time and temperature which were set in previous studies. In all cases, samples obtained after adsorption were filtered and then the residual concentration of Au (III) was determined by atomic absorption spectrometry using an atomic absorption spectrometer type Varian SpectrAA 280 FS. The adsorption capacity of the material, q (mg/g), was calculated using the following equation:

1 $${\rm{q}}=\frac{({{\rm{C}}}_{0}-{{\rm{C}}}_{{\rm{f}}})\,{\rm{V}}}{{\rm{m}}}$$

where: C_o_ – initial concentration of gold (III) from solution, (mg/L)

       C_f_ - the residual gold (III) concentration from solution, (mg/L)

       V - volume of solution, (L)

       m - mass of adsorbent material, (g)

### Gold recovery from exhausted material

Used adsorbent material to remove gold (III) from residual solutions after depletion contains considerable amounts of gold. For recovery of gold, depleted material was subjected to a thermo gravimetric analysis to determine the temperature at which its decomposition results in metallic gold. Thermal analysis was performed using the NETZCH STA 449C thermo gravimetric balance. Sample heating was performed in aluminium oxide crucibles at a rate of 10 K/min up to 1273 K in an air atmosphere. The decomposition of the sample was carried out at 873 K for 240 minutes at a heating rate of 5 K/min, using a controlled air oven (Nabertherm LHT407GN Furnaces). The sample obtained after decomposition was analysed by scanning electron microscopy (SEM) and X-ray energy dispersion (EDX) using the QUANTA FEG 250 microscope.

## Results and Discussions

### Characterization of the functionalized material

To prove that the polymeric support was functionalized with extractant molecules, was evidenced the presence of nitrogen atoms from glutamic acid into the functionalized material by recording the X-ray dispersion (EDX) spectra for the new produced adsorbent material. Obtained experimental data are presented in Table 1. Data depicted in Table 1 reveals the presence of a small amount of nitrogen (5.83%), specific for the NH_2_ group, which confirms the functionalization of the polymer support.

**Table 1:** Semi-quantitative analysis of the functionalized material.

Elem	Wt%	At%
C	68.59	74.26
N	5.83	1.70
O	25.58	24.04
Total	100.00	100.00

For highlighting the specific Amberlite XAD 7 polymer support groups and simultaneously the presence of the groups specific for the extractant molecules, FT-IR spectroscopy was used as groups identifying method.

**Figure 1:**
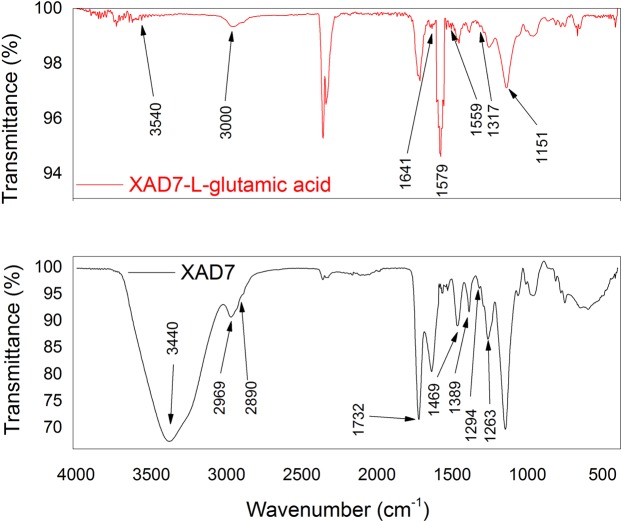
FT-IR spectra: Amberlite XAD7-glutamic acid.

The spectrums recorder for Amberlite XAD7 and for Amberlite XAD7 functionalized with glutamic acid are shown in Fig. 1.

From the FT-IR spectrums, it can be seen the presence of an adsorption band located around 3440 cm^−1^, band which is associated with the O–H group stretching vibration, at 2969 cm^−1^, 2890 cm^−1^, 1469 cm^−1^ and 1389 cm^−1^ there are specific vibrations for the aliphatic C–H bonds and at 1732 cm^−1^, 1263 cm^−1^ with shoulders at 1294 cm^−1^,1317 cm^−1^, and 1151 cm^−1^ occur vibrations specific for the C-O bond, all of these vibrations being specific to the Amberlite XAD7 support, most of them being powerful vibrations^27,28^.

At 3540 cm^−1^ there are specific vibrations for the NH_2_ group, around the wavelength of 3000 cm^−1^ there are vibrations specific to the OH groups. Also, the adsorption band located at 1641cm^−1^, is associated with the presence of stretching vibrations of C=O bond from the COOH dimmer group, or asymmetric vibrations of the C=O group from the COO^−^, specific for L-glutamic acid^1,2^.

It is also observed that instead of the vibration located at 1559 cm^−1^, the presence of two weak bands located at 1560 cm^−1^ and 1541 cm^−1^, which can be related with the functionalization of the polymeric support with the amino acidic specific. Also, into the spectrum of functionalized material can be observed the presence of an N-H specific peak located at 1579 cm^−1^, which can confirm the functionalization of the polymer support with the amino acid specific groups.

Withal, the functionalization of used polymeric support was confirmed by BET analysis (data are not presented in this paper), when was observed that the specific surface area of adsorbent material decreases after functionalization due to the presence of the extractant into the support pores. Another confirmation of material functionalization was obtained by comparing the adsorption capacities of unfunctionalized and functionalized material. With unfunctionalized material we get a maximum adsorption capacity of 1 mg Au (III)/g of adsorbent material and with functionalized material adsorption capacities were around 14 mg/g. Conclusion is we successfully produced a functionalized adsorbent material based on Amberlite XAD7 resin.

### Adsorption studies

#### Effect of pH

**Figure 2:**
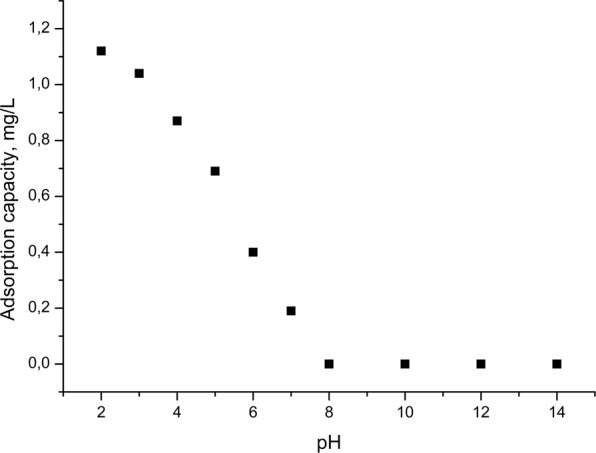
Effect of pH on adsorption Au(III).

One key parameter which controls adsorption process is represented by the pH at which the adsorption process is conducted. Figure 2 shows the effect of pH on the adsorption capacity of Au (III) on Amberlite XAD7-glutamic acid material at an initial concentration of gold ions of C_0_ = 5 mg/L, 1 hour contact time, and the temperature of 298 K. Studies have been conducted at a pH from 2 to14, by using 0.1 g of adsorbent material.

Data presented in Fig. 2 indicate that once the pH increases, the adsorption capacity of the material decreases, so that the adsorption process occurs with good results at pH < 4. At higher pH, the adsorption capacity drop is drastic and when pH value is higher than 8, the adsorption capacity is touching zero value, so the adsorbent material is no longer active for gold recovery. At low pH, when the pH of the solution was controlled by HCl, the amount of chloride in the solution was sufficiently high to favour the formation of the golden-chloro-anionic species which have been adsorbed by the protonated amino groups of the L-glutamic acid^29^. Moreover, the protonation of the amine group present in the studied material induces an electrostatic attraction on the anionic complex of the gold (III), increasing the number of free bonds available to the bonding of the metal ion. In case of acid solutions, the adsorption mechanism of Au (III) on Amberlite XAD7-glutamic acid is supposed to be an electrostatic attraction, but it can also be an ion exchange^2^. In the presence of chlorine ions, the interactions between the metal ion and the active centres can be presented as follows:

$$({\rm{R}}-{{\rm{NH}}}_{3}^{+}){{\rm{Cl}}}^{-}+{{\rm{AuCl}}}_{4}^{-}\leftrightarrow ({\rm{R}}-{{\rm{NH}}}_{3}^{+}){{\rm{AuCl}}}_{4}^{-}+{{\rm{Cl}}}^{-}$$

At pH > 4, the adsorption capacity decreases due to the fact that the number of absorbable species is much lower, with fewer free chlorine ions. This result is in concordance with the determined potential of zero charge (pzc) of the produced adsorbent material which has a value between 2 and 4.

Obtained results indicate that Au (III) adsorption takes place with high adsorption capacity only in the range of pH 1.0 to 4.0^2,30–33^. Literature data confirms that gold (III) recovery is performed with good efficiency at low pH, which represents an important parameter^1,31,34^.

### Influence of contact time and temperature

**Figure 3:**
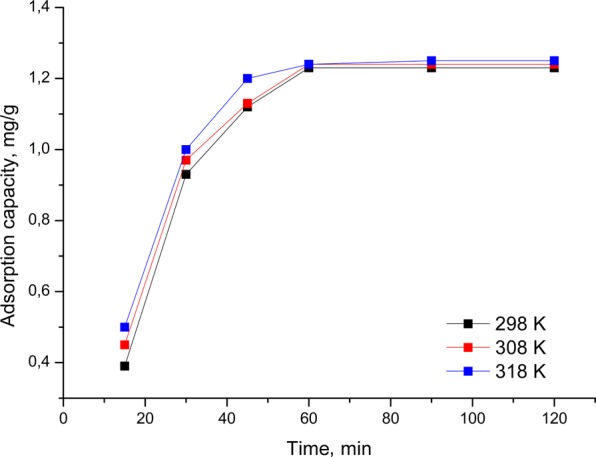
Effect of contact time and temperature onto gold (III) adsorption process.

The influence of contact time on the adsorption process of Au (III) on AmberliteXAD7 - glutamic acid, at three distinct temperatures, is shown in Fig. 3.

From the data presented in Fig. 3, which represents the effect of contact time and temperature on the adsorption process of gold (III) on the obtained material, it is observed that the material adsorption capacity increased with the increase of the contact time up to 60 minutes, after which the adsorption capacity remain constant, reaching a plateau. After 60 minutes, the largest amount of gold from used solution is adsorbed, the adsorption capacity being ~1.2 mg Au (III)/g of adsorbent material). It is also noted that the temperature positively influences the adsorption process, but this influence is not a significant one. Once the temperature increases, the adsorption capacity of the material increases, but economically it is not cost-effective. Thereafter, the studies were conducted at a temperature of 298 K and at a contact time of 60 minutes. Material performance can be attributed to the large contact surface required for adsorption processes.

### Effect of initial concentration

**Figure 4:**
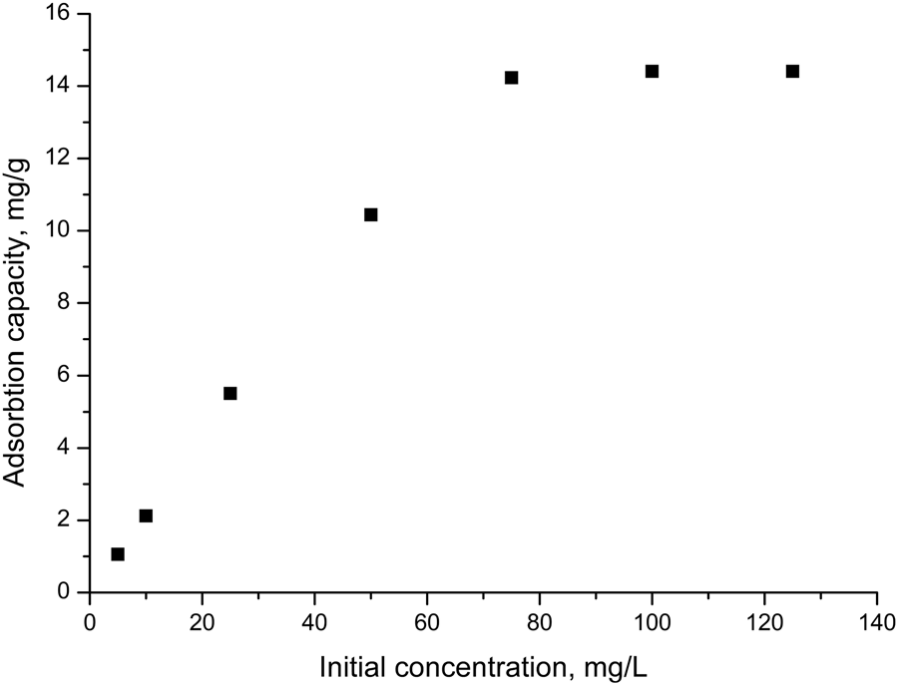
Effect of initial concentration of Au (III) onto adsorption process.

The effect of the initial concentration of the Au (III) solution on the adsorption process is shown in Fig. 4.

Analyzing data presented in Fig. 4 can observe that the adsorption capacity of produced adsorbent material increase with the increase of Au (III) initial concentration in solution, until the adsorbent material is saturated. Further increase of the initial concentration has no effect into the adsorption capacity which remains constant. Thus, the maximum adsorption capacity of the prepared adsorbent material is 14.23 mg Au (III)/g, for an initial Au (III) concentration of ~ 75 mg Au (III)/L.

### Adsorption isotherms

Au (III) adsorption mechanism on Amberlite XAD7-glutamic acid material was described by using Freundlich, Langmuir and Sips models. Langmuir isotherm is applied for homogeneous surface adsorption^35–37^. Non-linear expression of the Langmuir isothermal equation^38^ can be expressed as bellow:

2 $${{\rm{q}}}_{{\rm{e}}}=\frac{{{\rm{q}}}_{{\rm{\max }}}{{\rm{K}}}_{{\rm{L}}}\,{{\rm{C}}}_{{\rm{f}}}}{{1+K}_{{\rm{L}}}\,{{\rm{C}}}_{{\rm{f}}}}$$

where:

*q*_*e*_ - the maximum absorption capacity (mg/g)

*C*_*f*_ *-* the equilibrium concentration or final concentration of Au(III) in solution (mg/L)

*q*_*max*_ - Langmuir maximum adsorption capacity (mg/g)

*K*_*L*_ - Langmuir constant.

Freundlich isotherm can be applied for heterogeneous adsorption surfaces^36,39^. Non-linear form of the Freundlich isotherm equation^40^ is:

3 $${{\rm{q}}}_{{\rm{e}}}={{\rm{K}}}_{{\rm{F}}}\,{{\rm{C}}}_{{\rm{f}}}^{1/{{\rm{n}}}_{{\rm{f}}}}$$

where:

*q*_*e*_ - the maximum absorption capacity (mg/g)

*C*_*f*_ - the equilibrium concentration or final concentration of Au (III) in solution (mg/g)

*K*_*F*_ and *n*_*F*_ - the characteristic constants that can be related to the relative adsorption capacity of the adsorbent and the intensity of adsorption

Sips’ isotherm is a combined form of the two previously presented models. Its nonlinear form^41^ is the following:

4 $${q}_{e}=\frac{{q}_{s}\,{K}_{S}\,{C}_{e}^{1/{n}_{S}}}{1+{K}_{S}\,{C}_{e}^{1/{n}_{S}}}$$

where:

*q*_*S*_ *-* the maximum absorption capacity (mg/g)

*K*_*S*_ - constant related to the adsorption capacity of the adsorbent

*n*_*S*_- the heterogeneity factor

**Figure 5:**
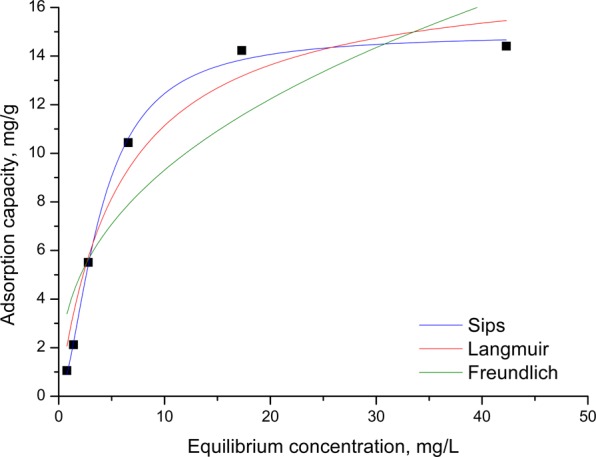
Adsorption isotherms of gold (III).

The experimental data, fitted using Eqs (2–4), are presented in Fig. 5 and the associated determined parameters are presented in Table 2.

**Table 2:** Adsorption isotherm parameters of Au (III) onto functionalized Amberlite XAD7 with glutamic acid.

	Parameters	XAD7-glutamic acid
Experimental values		
	*q*_m,exp_(mg/g)	14.23
Isotherm models		
Langmuir	*q*_L_ (mg/g)	17.6
	*K*_L_ (L/mg)	0.17
	R^2^	0.9557
Freundlich	*K*_F_ (mg/g)	3.75
	1/*n*_F_	0.39
	R^2^	0.8004
Sips	*q*_s_ (mg/g)	**14.9**
	*K*_s_	0.32
	1/*n*_s_	−0.71
	R^2^	**0.9972**

Based on data presented in Table 2 can observe that the highest regression coefficient R^2^ was obtained when obtained experimental data were modelled using Sips isotherm (0.9972), unlike the Langmuir isotherm (0.9557) and the Freundlich isotherm (0.8004). Thus, we conclude that the adsorption process of Au (III) on Amberlite XAD7-glutamic acid is best described by the Sips model. It is also observed that the studied material has a maximum experimental absorption capacity of 14.23 mg Au (III)/g and the theoretically established capacity after the experimental data were modelled using Sips isotherm, is 14.9, a value very close to the experimental one, being a confirmation that this model best describes the adsorption process of Au (III) on the studied material. These results reveal that the adhesion of Au (III) to Amberlite XAD7-glutamic acid is a heterogeneous process because the coefficient n_s_ > 1^42^.

Compared with other materials having adsorbent properties, the new produced material has a good adsorption capacity, unlike the TiO_2_ immobilized on silica gel nanomaterial with a maximum capacity of 3.56 mg Au (III)/g^43^ or 2-mercaptobenzothiazole-bonded silica gel having a capacity of 4.5 mg Au (III)/g^44^.

### Adsorption kinetics

The kinetics of gold (III) adsorption process on Amberlite XAD7-glutamic acid has been studied using two kinetic equations that could describe it: pseudo-first-order kinetic equation proposed by Lagergren and the pseudo-second-order kinetic equation proposed by Ho and McKay.

The pseudo-first-order equation can be expressed as such:

5 $$\frac{{{\rm{dq}}}_{{\rm{t}}}}{{\rm{dt}}}={{\rm{k}}}_{1}({{\rm{q}}}_{{\rm{e}}}-{{\rm{q}}}_{{\rm{t}}})$$

where: q_e_ and q_t_ are the adsorbed amounts of gold per unit mass of Amberlite XAD7-glutamic acid at equilibrium and time t respectively, and

k_1_ - is the rate constant for pseudo-first-order adsorption.

The q_t_ at different time values (t) can be determined by the following pseudo-first-order kinetic equation after integrating:

6 $$\mathrm{ln}({{\rm{q}}}_{{\rm{e}}}-{{\rm{q}}}_{{\rm{t}}})={{\rm{lnq}}}_{{\rm{e}}}-{{\rm{k}}}_{1}{\rm{t}}$$

The pseudo-second-order kinetic model can be presented with the following equation:

7 $$\frac{{{\rm{dq}}}_{{\rm{t}}}}{{\rm{dt}}}={{\rm{k}}}_{2}{({{\rm{q}}}_{{\rm{e}}}-{{\rm{q}}}_{{\rm{t}}})}^{2}$$

where k_2_ is the rate constant for the pseudo-second-order adsorption.

By linearizing this equation, we obtain:

8 $$\frac{{\rm{t}}}{{{\rm{q}}}_{{\rm{t}}}}=\frac{1}{{{\rm{k}}}_{2}{{\rm{q}}}_{{\rm{e}}}^{2}}+\frac{{\rm{t}}}{{{\rm{q}}}_{{\rm{e}}}}$$

Further, obtained experimental data were modelled using the linear form of the two considered kinetic models. The speed constant for the pseudo-first order model is determined from the linear representation of ln(q_e_ − q_t_) over time, and the speed constant for the pseudo-second-order model is estimated from the linear representation of the t/q_t_ function of time.

Based on the obtained values of constants and regression coefficients (R^2^), the kinetic model that best describes the adsorption process can be established.

**Figure 6:**
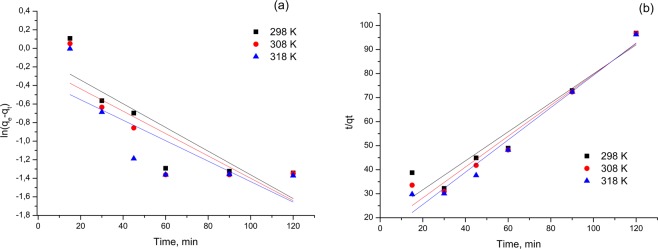
The kinetic mechanism that controls the gold (III) adsorption process onto Amberlite XAD 7 - glutamic acid, (**a)** Pseudo first order, (**b)** Pseudo second order.

Results obtained when the experimental data were modelled using considered kinetic models at considered temperatures are shown in Fig. 6 and associated kinetic parameters are shown in Table 3.

**Table 3:** Comparison of pseudo-first-order and pseudo-second-order rate constants and experimental q_e_ values.

Temperature (K)	Pseudo-first-order kinetics				Pseudo-second-order kinetics			
	q_e_ (exp) (mg/g)	k_1_ (min^−1^)	q_e_ (calc) (mg/g)	R^2^	q_e_ (exp) (mg/g)	k_2_ (g/mg min)	q_e_ (cal) (mg/g)	R^2^
298	14.23	0.0047	5.45	0.7626	14.23	36.74	11.82	0.9309
308	14.24	0.0061	5.28	0.7125	14.24	41.93	12.25	0.9561
318	14.25	0.0069	5.25	0.6372	14.25	47.46	12.25	0.968

In the case of the pseudo-first-order kinetic model, there are very different values between the calculated adsorption capacity (~5 mg/g) and the experimentally determined one (~14 mg/g).

Starting from the value of regression coefficient R^2^, it can be stated that the Au (III) adsorption process on Amberlite XAD7-glutamic acid behaves kinetically, like the pseudo-second-order kinetic model (R^2^ > 0.93). When the experimental data were modelled using pseudo-second-order model the calculated adsorption capacity had a value closed to the experimental determined one, being a confirmation that the studied process is better described by the pseudo-second-order model.

Activation energy (E_a_) value can provide information about the nature of the adsorption process, from physical or chemical point of view. The activation energy was calculated using relation:

9 $${{\rm{k}}}_{2}={\rm{A}}\,\exp (\frac{{{\rm{E}}}_{{\rm{a}}}}{{\rm{RT}}})$$

where:

k_2_ - is the pseudo-second-order rate constant of sorption (g/mg min),

*A* - is the Arrhenius constant which is a temperature independent factor (g/mg min),

*E*_a_ - is the activation energy of sorption (kJ/mol),

*R* - is the gas constant (8.314 J/mol K), and

*T* - is the absolute temperature (K).

**Figure 7:**
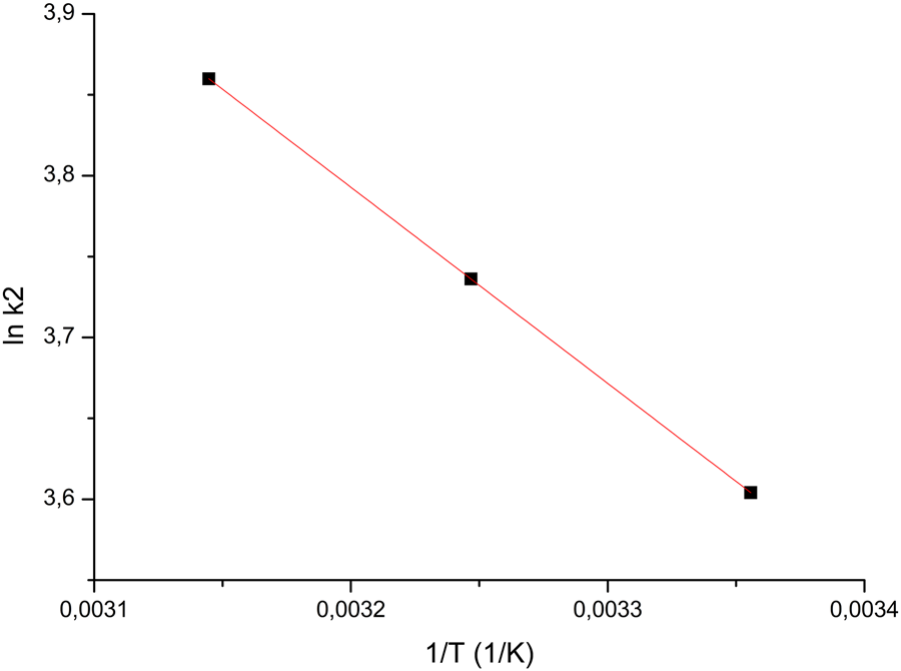
Relationship between lnk_2_ and 1/T for material.

From the graphical representation lnk_2_ = f (1/T) was determined the value of the ratio -E_a_/R (Fig. 7).

Based on obtained experimental data, the activation energy was calculated, this being 10.077,56 kJ/mol. Since the activation energy is greater than 4.2 kJ/mol, the adsorption process is a physical-chemical one, the attracting forces being more chemical than physical.

### Thermodynamics of the adsorption process

The effect of temperature on the Au (III) adsorption process on Amberlite XAD7-glutamic acid material was discussed above. Because, was observed that the adsorption capacity increases when the temperature increases, we can say that the studied adsorption process is an endothermic one. Specific thermodynamic parameters were calculated: free energy (ΔG°), free enthalpy (ΔH°) and free entropy (ΔS°) with the following relations:

10 $${\rm{\Delta }}G^\circ =-\,{{\rm{RTlnK}}}_{{\rm{c}}}$$

where

11 $${{\rm{K}}}_{{\rm{d}}}=\frac{{{\rm{C}}}_{{\rm{Ae}}}}{{{\rm{C}}}_{{\rm{e}}}}$$

12 $${\rm{so}}\,\mathrm{log}\,{{\rm{K}}}_{{\rm{d}}}=\frac{{{\rm{\Delta }}S}^{0}}{2.3\,{\rm{R}}}-\frac{{{\rm{\Delta }}H}^{0}}{2.303\,{\rm{RT}}}$$

where: *R* - is the gas constant,

*K*_d_ - is the equilibrium constant,

*T* - is the temperature (K),

*C*_Ae_ - is the equilibrium concentration Au (III) on adsorbent (mg/L), and

*C*_e_ - is the equilibrium concentration of Au (III) in the solution (mg/*L*).

**Figure 8:**
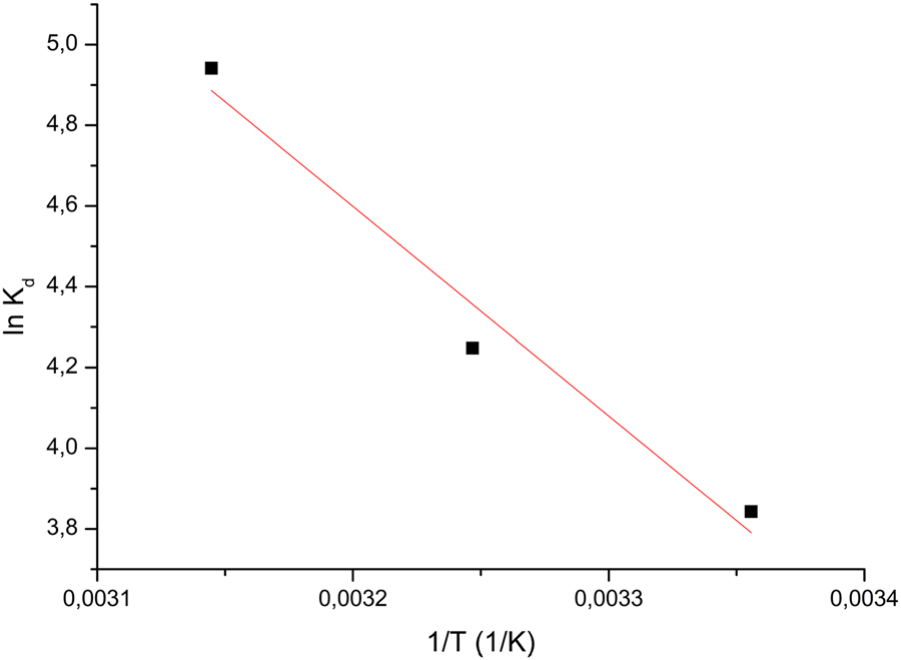
Van’t Hoff plots for the adsorption of Au (III) onto XAD7-glutamic acid.

Thermodynamic parameters calculated for Au (III) adsorption on the obtained material were evaluates from the slope of the straight line and ordered at the origin of the linear representation of lnK_d_ as a function of 1/T (Fig. 8). The values of ΔG°, ΔH° and ΔS° are shown in Table 4.

**Table 4:** Thermodynamic parameters for the adsorption of Au (III) onto Amberlite XAD7-glutamic acid.

T (K)	ΔH^0^ (kJ/mol)	ΔS^0^ (J/mol K)	ΔG^0^ (kJ/mol)	R^2^
298	15.43	55.8	−1.22	0.9718
308			−1.77	
318			−2.33	

Negative values of Gibbs free energy (ΔG°) and positive values of enthalpy (ΔH°) indicate that Au (III) adsorption on produced adsorbent material is a spontaneous and an endothermic process. The fact that with the temperature increase the free energy decreases indicates the existence of a small driving force, which confirms that the adsorption process is not significantly influenced by temperature, the adsorption capacity being almost the same regardless of the temperature. The positive value of standard entropy (ΔS^0^) indicates a decrease in free spaces at the solid-liquid interface during Au (III) adsorption on the material, suggesting also that the system presents disordered adsorption. Free enthalpy of less than 80 kJ/mol can be indicating that the studied adsorption process is a physical-chemical one.

In Table 5 is presented a comparison between the maximum adsorption capacities obtained for gold recovery when different materials were used as adsorbents. Based on data presented in Table 5 can observe that the new produced material (Amberlite XAD7 functionalized with L-glutamic acid) represents a useful adsorbent for gold recovery from diluted solutions.

**Table 5:** Comparison of maximum adsorption capacities obtained for different adsorbents.

Adsorbent	pH	Maximum adsorption capacities, q_max_ [mg/g]	Reference
Amberlite XAD 2000	2	12.3	^45^
Dowex M 4195	4	8.1	^46^
*Sargassum sp*.	2 to 10	0.17	^47^
*Turbinaria conoides*	2	0.18	^48^
*Fucus vesiculosus*	7	0.35	^49^
Ca-alginate beads	2	1.47	^50^
L-cysteine impregnated alginate capsules	5	1.51	^51^
Sulphuric acid cross-linked alginate powder	1	5.64	^52^
Thiourea modified alginate powder	—	6.40	^53^
Porous epichlorohydrin/ thiourea modified alginate (PETA)	1	1.97	^54^
Amberlite XAD 7	<4	1	Present study
Amberlite XAD 7-L-glutamic acid	<4	**14.2**	Present study

### Gold recovery from exhausted material

**Figure 9:**
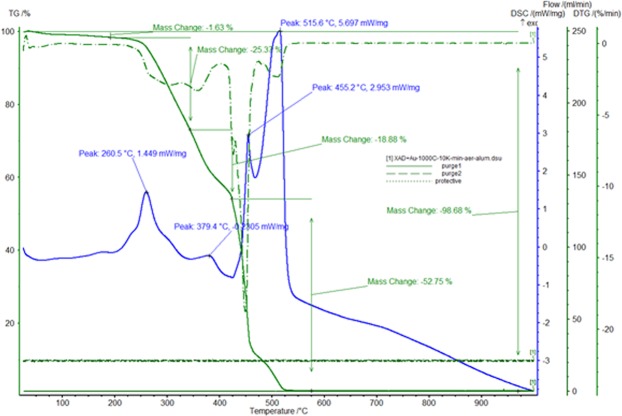
Thermogravimetric analysis of exhausted material in the air.

Thermogravimetric analysis performed in air for the exhausted adsorbent material used for Au (III) recovery from residual diluted solutions is shown in Fig. 9.

The analysis was necessary to determine the decomposition temperature of the material in order to recover gold in metallic form.

The graph shows that the decomposition process takes place in several stages. The first stage of decomposition is between 473 K and 623 K and has a mass loss of about 25%, being an exothermic process, probably because of the amino acid decomposition. The decomposition of the polymeric substrate in air takes place between 623 K and 823 K, with a mass loss of over 70%. This process is exothermic, complex, with several overlapping stages. The final residue represents approximately 1.3% of the initial sample mass, corresponding to ash and gold. As a result of this analysis, it was established that the thermal treatment of the exhausted material must be carried out in air atmosphere at 873 K. Sample heating should be done slowly for 240 minutes, at a rate of 5 K/min to obtain metallic gold and to remove the organic part of it.

**Figure 10:**
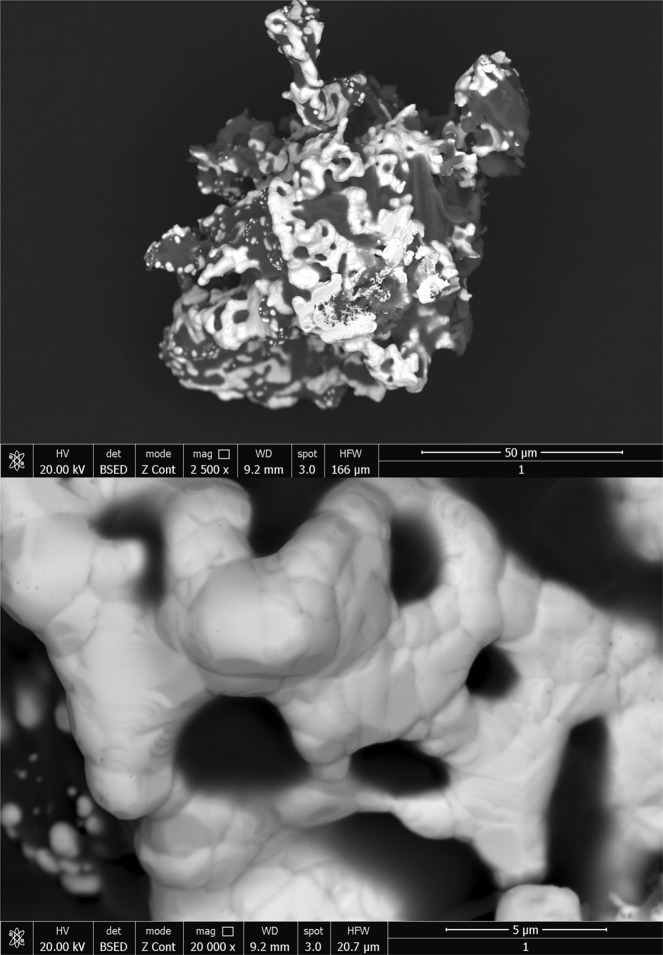
Scanning Electron Microscopy, SEM.

**Figure 11:**
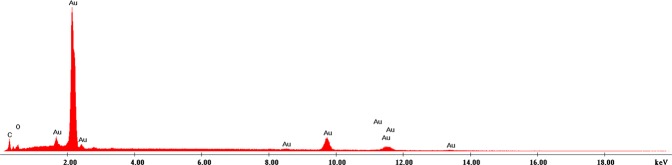
Energy Dispersive X-Ray Analysis (EDX).

The obtained sample after decomposition was analysed by electronic scanning microscopy - SEM (Fig. 10) and by X-ray dispersion (EDX) (Fig. 11).

Obtained SEM images provide information regarding particle morphology and the distribution of gold particles in the ash mass. There is a relative uniformity in the distribution of gold. The sample was also analysed by X-ray dispersion (EDX) (Fig. 11), and the chemical composition is presented in Table 6.

**Table 6:** Chemical composition after thermal decomposition of used adsorbent.

Elem	Wt.%	At%
C K	23.18	35.95
O K	39.20	45.64
NaK	8.59	6.96
SiK	1.10	0.73
P K	9.02	5.43
K K	9.19	4.38
AuL	9.72	0.92
Total	100.00	100.00

Based on the recorded EDX spectra has confirmed the presence of gold in the resulting ash after decomposition of the exhausted material. The other elements present into the material resulted after thermal decomposition are the elements specific for ash.

**Figure 12:**
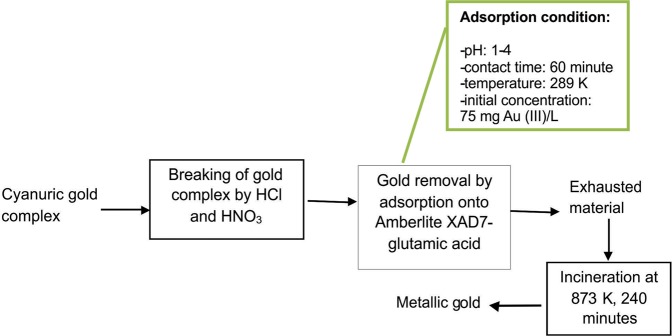
Proposed process for recovery metallic gold from waste dilute solution.

From presented data, it can be concluded that gold can be easily recovered from the exhausted material. Bellow, it is proposed a process of recovering metallic gold from waste cyanide solution through adsorption on an environmental friendly and relatively inexpensive material, Amberlite XAD7-glutamic acid, followed by thermal decomposition after depletion (Fig. 12).

The obtained gold can be reintroduced into specific technological processes.

## Conclusions

Experimental results obtained at the laboratory scale demonstrated that the new material obtained by the functionalization of the Amberlite XAD7 resin with active amino acid groups of L-glutamic acid (N and COOH) showed increased efficiency for the removal of Au (III) from diluted residual solutions resulted from cyanide electroplating baths. The maximum adsorption capacity of the material was 14.23 mg Au (III) per g of adsorbent material, for a maximum concentration of Au (III) of 75 mg/L. The required contact time was 60 minutes.

The mechanism of the process involved the breaking of cyanuric gold complex by a mixture of HCl and HNO_3_, obtaining ${{\rm{AuCl}}}_{4}^{-}\,$. It is worth mentioning the fact that the process proceeds with maximum efficiency in the pH range 1 ÷ 4. At the same time, proposed adsorption mechanism is supported by performed kinetic, thermodynamic and equilibrium studies. Thus, the adsorption process is subject to pseudo-second-order kinetics, and the isotherm that covers in the best way the adsorption process, is Sips one. Studied adsorption process is spontaneous, and the adsorption is accomplished by physical-chemical interactions between the metal ion and the active centres of the material.

Proposed process of recovering metallic gold from exhausted adsorbent material is another target of this study. By incinerating the exhausted material at 873 K metallic gold is obtained, which can then be used in various industrial fields such as: electronics, medicine, jewellery industry, chemical industry, as a catalyst, for obtaining materials with anticorrosive properties etc.
